# Role of the Proportional Odds Assumption for the Analysis of Ordinal Outcomes in Neurologic Trials

**DOI:** 10.1212/WNL.0000000000214146

**Published:** 2025-09-25

**Authors:** Yongxi Long, Eveline J.A. Wiegers, Bart C. Jacobs, Ewout W. Steyerberg, Erik W. van Zwet

**Affiliations:** 1 Biomedical Data Sciences, Leiden University Medical Center, the Netherlands;; 2 Department of Neurology, Erasmus MC, Rotterdam, the Netherlands;; 3 Department of Neurology, Department of Immunology, Erasmus MC, Rotterdam, the Netherlands; and; 4 Julius Center for Health Sciences and Primary Care, University Medical Center Utrecht, the Netherlands.

## Abstract

Ordinal scales are categories ordered by their clinical preference, such as the modified Rankin Scale and Glasgow Outcome Scale Extended. They are widely used as key outcome measures in neurologic trials. Compared with binary outcomes, ordinal scales allow a more detailed assessment of the effect of the treatment and provide more statistical power. Typically, the proportional odds (PO) model (or “shift analysis”) is used to quantify the treatment effect in a common odds ratio (cOR) and to test the null hypothesis that the treatment has no effect. Clinical researchers and trialists may worry that the PO assumption will not hold and therefore decide to dichotomize the ordinal scale into a binary outcome. Here, we explain that for the purpose of testing the presence of a treatment effect, it is irrelevant whether the PO assumption holds. In fact, pretesting this assumption to decide which test to use for the treatment effect is invalid and can inflate the type I error probability. Although the PO assumption is not relevant for testing, it is definitely relevant for the purpose of describing and summarizing the treatment effect. We suggest a simple graphical check of the PO assumption as more informative than formal testing. If we are satisfied that there is no substantial violation of the PO assumption, it is reasonable to summarize the treatment effect into a single number such as the cOR. Otherwise, no single-number summary measure provides a faithful representation. We illustrate these practical considerations with 3 neurologic trials: the Endovascular Therapy in Acute Anterior Circulation Large Vessel Occlusive Patients with a Large Infarct Core (ANGEL-ASPECT) trial and the Multicenter Randomized Clinical Trial of Endovascular Treatment for Acute Ischemic Stroke in the Netherlands ([MR CLEAN], which investigated endovascular therapy in stroke) and the RESCUEicp trial (which investigated decompressive craniectomy in traumatic brain injury). We conclude with recommendations for the statistical workflow. Undue concerns about the PO assumption should not deter researchers from using ordinal scales in neurologic trials. We provide an R package *CORPlot *to facilitate the graphical check of the PO assumption.

## Introduction

Ordinal scales such as the modified Rankin Scale (mRS^1^) and the Glasgow Outcome Scale Extended (GOS-E^2^) are widely used for outcome assessment in clinical trials in neurologic diseases. Compared with binary outcomes, ordinal scales allow a more detailed assessment of the effect of the treatment on the functional status of patients and provide more statistical power when the treatment results in a general shift toward better (or worse) functional status.^3-7^

We recently reviewed the statistical practice of analyzing ordinal outcomes in current trials of acute neurologic diseases.^8^ One notable finding was that ordinal outcomes were dichotomized in about one-third of the included studies. Dichotomization results in a loss of statistical power when the underlying effect of the treatment is a global shift to better outcome categories.^9,10^ Irrespective of the effect pattern, dichotomization always causes a loss of information because the original scale cannot be recovered after dichotomization. The loss of information is especially problematic if the treatment effect is bidirectional, as in the European Cooperative Acute Stroke Study trial where patients on IV alteplase had both more good outcomes and more poor outcomes than control patients.^11^ Dichotomizing an ordinal outcome has been motivated by the clinical meaningfulness of a particular cut-point, which depends on the research question, mechanism of the intervention, and patient case-mix.^12^ We stress that even if the primary analysis is based on dichotomization, it remains important to explore the full ordinal scale. This aligns with the recommendations of the US Food and Drug Administration.^13^ Ordinal methods are powerful and informative, but they continue to be underused.

The proportional odds (PO) model^14^ is commonly used for the statistical analysis of ordinal outcomes. The model can be understood by considering the odds ratios resulting from dichotomizing the ordinal scale at all possible cut-points. The PO assumption states that these cumulative binary odds ratios are all equal. This means that the treatment results in a uniform shift across all the categories of the ordinal scale. If that is indeed the case, then it is reasonable to summarize the overall treatment effect as the average of the observed binary odds ratios. This summary is known as the “common odds ratio” which is often reported as the key measure of treatment effect.

Concerns about the validity of the PO assumption might be an important impediment to the use of ordinal scales.^8,15^ Some trials had planned an ordinal analysis by the PO model but switched to dichotomization^16^ when a statistical test suggested a violation of the PO assumption. Researchers also sometimes replace the test of the common odds ratio (cOR) from the PO model with the Mann-Whitney *U* test if the PO assumption seems violated.^17^

The aim of this study was to clear misconceptions around the PO assumption. The core message is 2 fold. First, we clarify that for the purpose of testing the null hypothesis of no treatment effect, it is irrelevant whether the PO assumption holds. In other words, even when the PO assumption does not hold, it is still perfectly valid to test the null hypothesis that the treatment has no effect by fitting the PO model and testing if the cOR is equal to 1. In fact, we will demonstrate that checking the PO assumption to decide which statistical test to use for the treatment effect is invalid because it inflates the type I error probability. Second, although the PO assumption is not relevant for testing, it is highly relevant for describing and summarizing the treatment effect. We recommend that researchers perform a graphical check of the PO assumption either by using Grotta bars^18^ or by plotting the cumulative binary odds ratios which we will demonstrate in the next section. We believe such graphical checks are more informative than formal hypothesis testing of the PO assumption because these tests often lack sufficient statistical power. Only if we are satisfied that there is no substantial violation of the PO assumption, then we should consider summarizing the treatment effect into a single cOR. We illustrate these considerations with 3 neurologic trials: the MR CLEAN trial^19^ and the ANGEL-ASPECT trial^20^ investigating endovascular therapy in patients with stroke, and the RESCUEicp trial investigating decompressive craniectomy in patients with traumatic brain injury.^21^

Besides the cOR, several other single-number effect sizes are available for ordinal outcomes. These include the various “win statistics,” such as the probabilistic index, net benefit, win odds, and win ratio.^22-24^ These effect sizes are based on comparing all participants on active treatment with all controls, and deciding who “wins” for having a better outcome condition. The win statistics are closely related to the Mann-Whitney *U* test. Similar to the cOR, these summary statistics are appropriate when the treatment causes a uniform shift across all levels of the ordinal scale. If there is a substantial violation of the PO assumption, then any single-number summary of the treatment effect is an oversimplification.

Finally, we discuss the role of attaching utilities to ordinal outcomes.^25^ Utilities are context-dependent and therefore to some extent subjective, but they add an additional layer of information.^26,27^ The advantage of utilities is that they provide the basis for rational decision-making.^28^

## Methods

### Statistical Checklist

**Figure 1:**
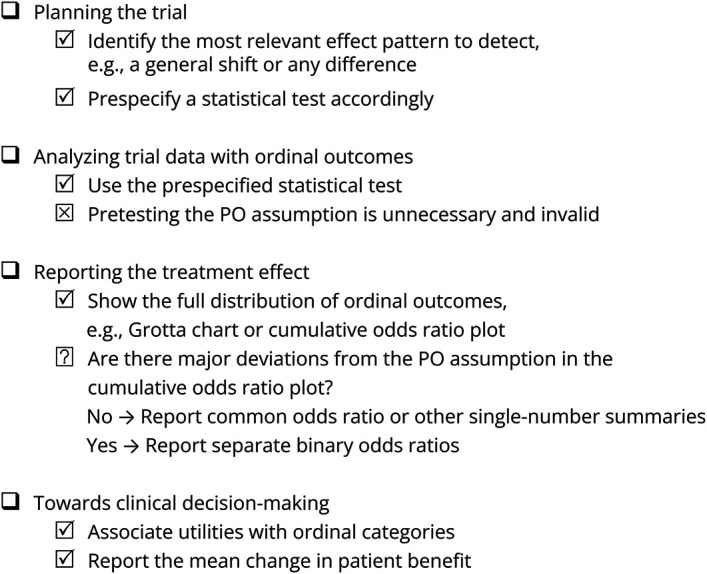
Author Checklist Statistical checklist for ordinal outcome analysis in neurologic trials.

We summarize our recommendations into a practical checklist presented in Figure 1. Each point will be elaborated in the following corresponding section. We aim to explain statistical concepts in words with real-world trial examples and straightforward simulation studies.

Technical details are referred to in relevant places and can be found in the online Statistical Appendix (eMethods). All simulation studies are recorded in the R code supplement (eAppendix1) for reproducibility, and the codes for producing tables and figures are also included (R Statistical Software v4.3.1; R Core Team 2021).

### The PO Model

The most widely^8,15^ used statistical model for analyzing ordinal outcomes in neurologic trials is the PO cumulative logistic model or PO model. This modeling approach is sometimes called a “shift analysis.”

For a binary outcome, logistic regression is typically used to estimate the odds ratio of the event of interest. For an ordinal outcome with K categories, there are K-1 ways to dichotomize it into a binary outcome. The PO assumption means that the associated K-1 binary odds ratios are assumed to be all equal so that there is only 1 cOR left. This cOR is estimated as a weighted average of the observed binary odds ratios for the K-1 dichotomizations.

Another way to think about the PO assumption and the PO model is to imagine an underlying (or latent) continuous outcome that has been categorized to produce the ordinal outcome. For a more careful discussion, we refer to Section 1 of the Statistical Appendix (eMethods). The PO assumption means that we assume that the treatment shifts the mean of the latent outcome without changing its shape.^29^

**Figure 2:**
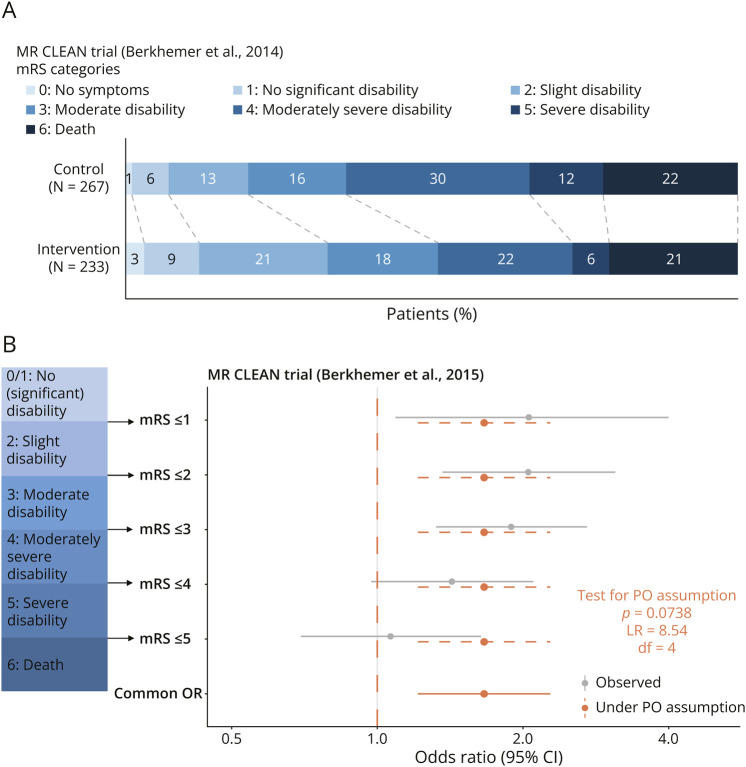
MR CLEAN Trial MR CLEAN trial investigated endovascular therapy in patients with acute ischemic stroke. (A) Grotta bars. The experimental treatment (endovascular therapy) resulted in a “shift” effect compared with the control (usual care). (B) Cumulative odds ratio plot. The plot shows 5 observed cumulative binary odds ratios with 95% CIs for each cut-point of the ordinal mRS outcome. The orange dashed lines show the common odds ratio based on the proportional assumption. *p* Value for the proportional odds assumption (along with associated test statistic and degrees of freedom) is from the likelihood ratio test.

As an example, we consider the MR CLEAN trial which compared endovascular therapy with usual care in patients with acute ischemic stroke. The primary outcome measure was the mRS at 90 days.^19^ Figure 2 panel A presents the Grotta bars illustrating the percentages for each outcome category in the control and intervention groups. We observe a general shift toward lower (better) categories. The mRS has 7 outcome categories, but due to the small sample size, we combined categories 0 (no symptoms) and 1 (no significant disability) for illustration purposes in Figure 2B. Given 6 outcome categories, there are 5 separate binary odds ratios which we show in gray together with their CIs. Assuming the true binary odds ratios are actually all the same (i.e., making the PO assumption) results in 1 cOR which we show in orange.

## Testing the Treatment Effect

We can assess the null hypothesis that the treatment has no effect by testing if the cOR from a PO model is equal to 1. Perhaps surprisingly, this test remains valid whether the PO assumption holds. A statistical test is defined to be valid if it properly controls the type I error rate at the prespecified significance level. In other words, if the null hypothesis is true and we want the type I error to be controlled at 5%, then a valid test should not reject with probability more than 5%. Under the null hypothesis of no treatment effect, the outcome distributions in both groups are identical. Therefore, all cumulative binary odds ratios are equal to 1. Thus, the PO assumption automatically holds, and there is no need to check it.

**Figure 3:**
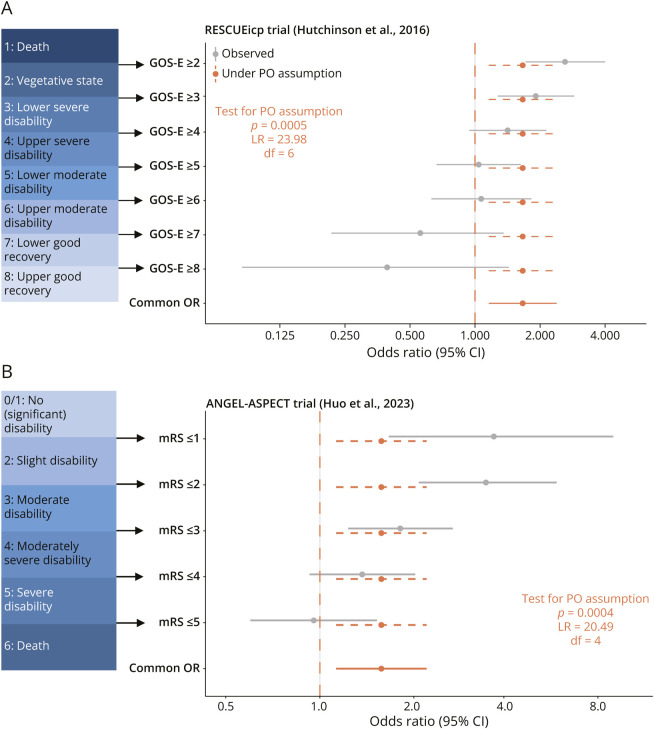
Cumulative Odds Ratio Plot Cumulative odds ratio plots for the (A) RESCUEicp trial (Hutchinson et al., 2016) and (B) ANGEL-ASPECT trial (Huo et al., 2023). Cumulative binary odds ratios at each cut-point are shown together with the common odds ratio. *p* Value for the proportional odds assumption (along with associated test statistic and degrees of freedom) is from the likelihood ratio test.

Whether the PO assumption holds (i.e., whether the treatment causes a uniform shift) does affect the statistical power of the test, which is the probability of obtaining a statistically significant result when there is truly an effect. Testing if the cOR is equal to 1 in the PO model has high power to detect a uniform shift in the ordinal categories. On the other hand, the test has very little power to detect changes in the categories that leave the cOR approximately equal to 1. This can happen, for example, when a beneficial effect for patients in some categories is offset by a harmful effect for patients in other categories, as was found for the RESCUEicp trial (Figure 3A).

**Figure 4:**
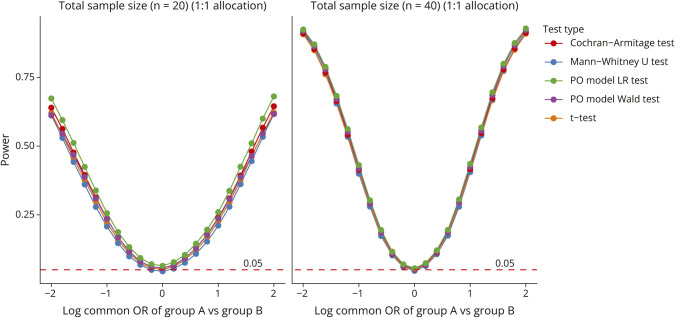
Power Comparison Comparison of power among the Cochran-Armitage test for trend/linear-by-linear association test, 2-sample independent Mann-Whitney *U* test, likelihood ratio (LR) test from the PO model, Wald test from the PO model, and 2-sample independent *t* test. The ordinal distribution of the treatment group varies from right skewed to left skewed, while the ordinal categories of the control group are equally distributed. This construction makes the log common odds ratio of treatment vs control vary from negative to null then to positive. PO = proportional odds.

There are many alternatives for the test of the cOR from the PO model, such as the Mann-Whitney *U* test, the Cochran-Armitage test for linear trend, and even the 2-sample sample *t* test, where we represent the ordinal categories by sequential numbers. Since these tests are all targeted at detecting a general shift, they have nearly identical statistical power. In Figure 4, we illustrate a test of an ordinal outcome with 5 levels between 2 groups. In the control group, the categories are all equally likely, while in the treatment group, we vary the distribution from right-skewed to left-skewed. Even with very small sample sizes, the power of the tests remains quite similar across a series of shift effects that vary from a negative trend to a positive trend (Simulation 1, eAppendix1).

The Mann-Whitney *U* test, in particular, is sometimes viewed as an alternative to the cOR test in case the PO assumption does not seem to hold.^17,20,30^ This is misguided as the validity of the cOR test does not depend on the PO assumption. In theory, the result of the 2 tests will be very similar.^31^

**Figure 5:**
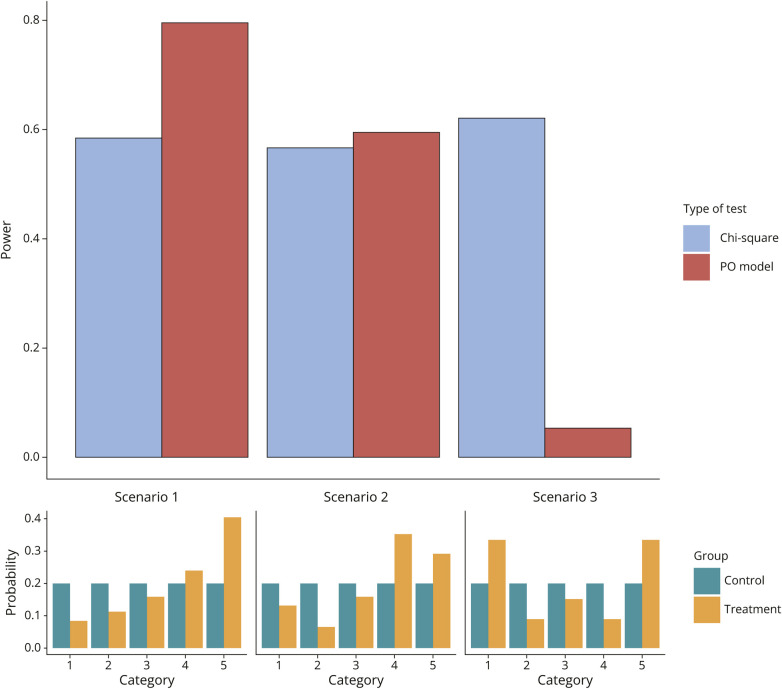
Shift vs Nonshift Test Scenario 1: an exact shift effect. The common odds ratio (cOR) is 2.7. The PO model (likelihood ratio test) has superior power over the χ^2^ test. Scenario 2: a semishift effect such that the positive trend is larger in categories in the middle than at the ends. The cOR is 2.2. The PO model and the χ^2^ test have similar power. Scenario 3: a nonshift effect with opposite trends. The cOR is 1.0. The χ^2^ test has superior power over the PO model. PO = proportional odds.

There are also various tests that broadly target *any* difference between 2 treatment groups, such as the χ^2^ test. The price to pay for this generality is reduced power against pure shift effects. We illustrate this trade-off in Figure 5 where we contrast 3 scenarios. In each of these cases, the 5 ordinal outcomes are equally likely under the control treatment. In scenario 1, the treatment causes a uniform shift across all levels of the 5 categories. The PO assumption is exactly satisfied, and the test of the cOR is more powerful than the χ^2^ test. In scenario 3, the situation is reversed. The treatment effects are toward opposite directions, so the PO assumption is not satisfied. The cOR is still mathematically well-defined (it is an average of the cumulative binary odds ratios^14^), and it is exactly 1. Now, the χ^2^ test is more powerful than the test of the cOR which has only 5% power. In scenario 2, there is a largely positive trend although the PO assumption does not hold. In this case, the 2 tests have approximately equal power (Simulation 2, eAppendix1).

We can demonstrate the power differences between the test of the cOR from the PO model (which focuses specifically on shifts) and the χ^2^ test (which focuses more broadly on any changes) in the 3 example neurologic trials. In the MR CLEAN trial, the treatment effect is approximately a uniform shift (Figure 2), so we expect the test from the PO model to be more powerful than the χ^2^ test (Table). The ANGEL ASPECT trial and the RESCUEicp trial (Figure 3) resemble the scenario 3 in Figure 5 where the effect is not a uniform shift. The *p* value from the PO model is much larger than the *p* value from the χ^2^ test (Table).

**Table:** *p* Values From the PO Model (Likelihood Ratio Test) vs *p* Values From the χ^2^ Test for the MR CLEAN Trial, the ANGEL-ASPECT Trial, and the RESCUEicp Trial

Trial	*p* Value from the PO model	*p* Value from the χ^2^ test
MR CLEAN	0.0015 (LR = 10.07, df = 1)	0.0028 (χ^2^ = 19.97, df = 6)
ANGEL-ASPECT	0.0035 (LR = 8.53, df = 1)	0.000035(χ^2^ = 28.07, df = 5)
RESCUEicp	0.0058 (LR = 7.62, df = 1)	0.000036 (χ^2^ = 30.19, df = 6)

Abbreviation: PO = proportional odds.

Note that in the case of the χ^2^ test for the ANGEL-ASPECT trial, mRS category 0 and 1 were merged, and for RECUEicp trial, GOS-E categories 7 and 8 were merged because of small numbers.

In summary, for the comparison of ordinal outcomes, different types of tests have different power under different treatment effect patterns. Obviously, it is not valid to cherry-pick the smallest *p* value. The test should be specified before seeing the data. Alternatively, we can try out various tests for different potential treatment effect patterns, but then we need to do a multiple test correction. In our review of ordinal outcome analysis in neurologic trials, we found many cases where the statistical analysis plan included performing a pretest for the PO assumption, and then deciding the test for the treatment effect depending on the result.^16,17,20,21,30,32^ This is probably meant in a similar spirit as checking the assumption of normality of a continuous outcome and then deciding between the *t* test and some nonparametric alternative (such as the Mann-Whitney *U* test). However, pretesting the PO assumption is not only unnecessary, but it is actually invalid. It is especially problematic if we use the pretest to decide between a test for detecting a global shift and the χ^2^ test. This essentially boils down to picking the smallest *p* value which will increase the probability of a type I error.

To demonstrate the inflation of the type I error, we conducted a small simulation experiment. We assume 5 outcome categories with equal probabilities of 1 of 5 in both groups. The null hypothesis is true, so the tests should reject the null hypothesis in not more than 5% of the cases. Now, we compare 3 strategies for testing: (1) always use the common odds test from the PO model, (2) always use the χ^2^ test, and (3) a hybrid strategy where we first test the PO assumption at alpha level 0.05. We find that the hybrid strategy inflates the type I error probability by about 60% (from nominal 0.05 to 0.08, Simulation 3, eAppendix1).

## Reporting and Interpreting the Treatment Effect

Whether the PO assumption holds does not affect the validity of the test of the cOR, but it does affect how the treatment effect should be summarized and interpreted. If there does not seem to be any substantial violation of the PO assumption, the cOR with the associated CIs can effectively summarize the treatment effect as a general positive or negative shift across the ordinal scale. However, when the PO assumption is clearly violated, no single-number summary measure is a faithful representation of the heterogeneous treatment effect. A cumulative odds ratio plot (such as Figures 2B and 3) provides a clear visual comparison between the observed treatment effects with and without the PO assumption. We provide an R package, *CORPlot,* for creating this type of plot (https://github.com/Yongxi-Long/CORPlot).

In the MR CLEAN trial, the cumulative odds ratio plot (Figure 2B) does not suggest substantial deviation from the PO assumption, and the authors decided to summarize the treatment effect into a single cOR of 1.67 (95% CI 1.21–2.28). It is also possible to perform a formal statistical test of the PO assumption. In this case, the likelihood ratio test resulted in a *p* value of 0.07. However, we caution against interpreting such a nonsignificant *p* value as proof that the PO assumption holds. After all, the absence of evidence is not evidence of absence.^33^ Moreover, clinical trials are powered for the analysis of the primary outcome, so tests for violations of the PO assumption will often have insufficient power. Therefore, we recommend a visual inspection of the binary odds ratios to judge the appropriateness of the cOR as an overall summary.

As examples of violation of the PO assumption, we present the ANGEL-ASPECT trial^20^ which investigated endovascular therapy in patients with stroke and the RESCUEicp trial^21^ which investigated decompressive craniectomy in patients with traumatic brain injury. We show the cumulative odds ratio plots for the 2 trials in Figure 3. We also performed formal statistical tests which rejected the PO assumption in both cases. In the case of the ANGEL-ASPECT trial (Figure 3B), the cOR is (significantly) larger than 1 and the data are compatible with a generally positive effect across all categories, even if binary odds ratios are not all the same. We could argue that the cOR can still summarize the overall beneficial effect of the treatment. In the case of the RESCUEicp trial (Figure 3A), the cOR is also (significantly) larger than 1, again indicating a net positive effect. However, there is some indication that the intervention (craniectomy) increased the proportions of patients in a vegetative state or with severe disability. Patients who underwent surgery were also less likely to achieve good recovery status compared with usual care. Thus in this case, reporting only the cOR would be a serious oversimplification and potentially misleading for clinical practice.

As we mentioned in the Introduction, the treatment effect on an ordinal scale can also be expressed in the so-called win statistics.^34,35^ It may be argued that their interpretation is more straightforward when the PO assumption is violated because a win/loss/tie is always clearly defined. Ultimately, however, they suffer from the same drawback as the cOR, namely, that a single number cannot faithfully represent a heterogeneous effect. We provide additional details about win statistics in Section 2 of the online Statistical Appendix (eMethods).

In conclusion, we recommend always reporting the full distributions of the outcome categories in both groups and showing either the Grotta bars (Figure 1A) or the cumulative odds ratio plot (Figures 2B and 3) so that readers may judge for themselves whether a single-number summary of the treatment effect is appropriate.

## The Treatment Effect in Utility

Changes on an ordinal scale can be mapped to more patient-centered effects that integrate quality of life or monetary costs. This is performed by attaching numerical utilities to each category of the ordinal outcome. Utilities are a set of numerical values that reflect the desirability or preference of each ordinal category from the perspective of patients, caregivers, or society. These values typically range from 0 (representing death or the worst health state) to 1 (perfect health), although negative values are sometimes used to represent states considered worse than death.^25,26^ The treatment effect can then be expressed as a (standardized) difference of mean utilities between the treated and control groups. To be clear, we do not promote the utility-weighted ordinal scale as an alternative to the ordinal scale as the primary endpoint, rather, we see the utilities as a supplementary step to incorporate more information. There are several advantages to consider utilities. First, they can address the differential clinical relevance of outcome categories. We illustrate this point by a comparison between ordinal analysis and utility analysis in Section 3 of the Statistical Appendix (eMethods). Second, utilities provide the information that is required for rational decision-making and cost-effectiveness analysis.^36^

**Figure 6:**
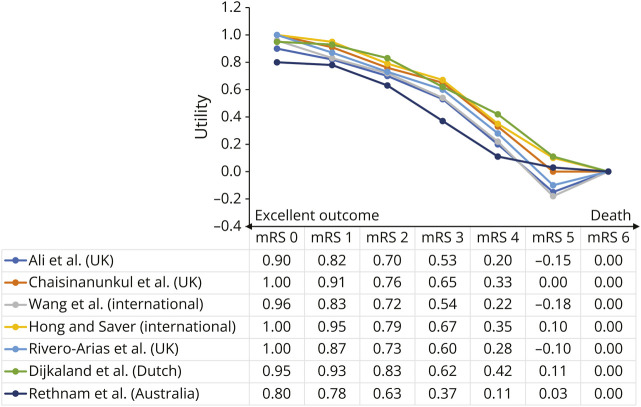
Utility-Weighted mRS Utility values for each mRS category from 7 studies. Multiple versions of utility-weighted mRS across countries were reported by Ali et al., 2017. mRS = modified Rankin Scale.

Utilities are context-dependent and therefore to some extent subjective (Figure 6). Different derivation methods, country-specific or region-specific value sets, quality-of-life instruments, and patient population characteristics can yield varying utility values for the same ordinal scale.^37^ The presence of subjectivity might be practically justified in this setting because it is both expected and reflective of real-world differences in how patients, clinicians, and caregivers perceive the value of specific health states.^36^ The variability of utility-weighted ordinal scales can be addressed, either by a well-informed choice of the value set or by considering multiple sets.

## Discussion

Compared with binary outcomes, ordinal scales offer a more nuanced evaluation of a treatment's effect on a patient's functional status. In addition, ordinal scales typically enhance statistical power, thereby reducing the necessary sample size. Recently, we reviewed the statistical methods used to analyze ordinal outcomes in recent trials involving acute neurologic diseases.^8^ We found that ordinal scales are sometimes dichotomized for the primary outcome,^8,15^ thus negating the advantages of ordinal over binary outcomes. This has prompted this study. We recommend the following workflow which we summarize in Figure 1.

First, we must decide on the statistical test of the primary outcome before seeing the data. If the experimental treatment is expected to result in a general shift toward more favorable outcome categories, then we recommend a statistical test that is targeted to detect such a shift. Popular choices are the cOR test or the Mann-Whitney *U* test. If one expects a more differentiated effect of the treatment, then the χ^2^ test may be more powerful.

Second, the validity of these tests does not depend on whether the PO assumption holds. Therefore, it is not necessary to pretest this assumption. In fact, deciding the test of the treatment effect on the basis of the test of the PO assumption invalidates it.

Third, it is important to report the full distribution of ordinal outcomes for both groups. We recommend inspecting the associated Grotta bars or cumulative odds ratio plot to assess the effect across the levels of the ordinal scale. The cumulative odds ratio plot is especially useful for a comprehensive assessment of the PO assumption. It is also possible to formally test the PO assumption. However, as trials are powered for the primary treatment effect, the statistical power to detect violations of the PO assumption is often insufficient. If the PO assumption seems doubtful, then any single-number summary of the treatment effect risks oversimplification and could be misleading for clinical practice.

Fourth, we recommend considering associating utilities with the ordinal categories as a step toward clinical decision-making and cost-effectiveness analysis. Translating an ordinal to a numerical scale might be subjective to some extent, but this is offset by the practical usefulness of utilities.

In conclusion, ordinal outcomes are informative but remain underused in neurologic trials. Concerns about potential violations of the PO assumption should not discourage researchers from their use. For testing the null hypothesis that the treatment has no effect, it is not necessary to check the PO assumption. We do recommend graphical assessments of the PO assumption to decide whether the effect of the treatment can be faithfully represented by a single-number summary.

## Glossary

cOR: common odds ratio
mRS: modified Rankin Scale
PO: proportional odds
